# GW117: A novel serotonin (5‐HT_2C_) receptor antagonist and melatonin (MT_1_/MT_2_) receptor agonist with potential antidepressant‐like activity in rodents

**DOI:** 10.1111/cns.13630

**Published:** 2021-03-01

**Authors:** Nana Gao, Wei Zheng, Tiliwaerde Murezati, Wei Gu, Xiaorong Li, Zengliang Jin

**Affiliations:** ^1^ Department of Gastrointestinal Surgery and Clinical Nutrition Beijing Shijitan Hospital,Captial Medical University Beijing China; ^2^ Department of Pharmacology School of Basic Medical Sciences Capital Medical University Beijing China; ^3^ Beijing Guangwei Pharmaceutical Technology Co., Ltd Beijing China

**Keywords:** 5‐HT_2C_ receptor, antidepressant, GW117, melatonin receptor

## Abstract

**Aims:**

To evaluate the antidepressant‐like effect of compound GW117 in rodents using in vitro binding and uptake assays as well in vivo behavioral tests.

**Methods:**

We investigated the target profile of GW117 using [^35^S]‐GTPγS and [^3^H]PIP binding. Using the forced swimming test and chronic unpredictable stress in rats, tail suspension test in mice and rats, and learned helplessness model in mice, we further revealed the antidepressant‐like and anxiolytic‐like effects of GW117.

**Results:**

The current study suggests that GW117 displays serotonin 2C (5‐HT_2C_) receptor antagonist and melatonin type 1 and 2 (MT_1_/MT_2_) receptor agonist properties, as well as evident antidepressant and anxiolytic effects.

**Conclusion:**

These data suggest that GW117 is probably a potent antidepressant.

## Graphical abstract

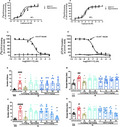

## 1 INTRODUCTION

Major depressive disorder (MDD) is one of the common psychiatric disorders, which affects more than 300 million persons of all ages (WHO, 2017). Indeed, MDD is a major contributor to the global burden of disease and a leading cause of disability worldwide due to the associated high rates of suicide and increased risk of comorbidity. Currently, monoamine‐based pharmacotherapies and psychobehavioral therapies are the main interventions.^1^, ^2^ The first‐line medications for MDD selective 5‐hydroxytryptamine reuptake inhibitors (SSRIs) that block the 5‐hydroxytryptamine transporter protein are also widely used psychiatric drugs for many other psychiatric disorders currently, including anxiety disorders, panic disorders, and bipolar disorder. Despite widespread use of these drugs, there are challenges in terms of their efficacy and side effects,^3^ which cognitive dysfunction, sexual dysfunction, sleep disorders, and weight gain and it often require 6–8 weeks to achieve effectiveness.^4^, ^5^ Growing evidence suggests that several side effects of SSRIs, especially anhedonia, apathy, and extrapyramidal motor symptoms, affect the behavioral and emotional efficacy of SSRIs.^6^, ^7^, ^8^, ^9^, ^10^, ^11^ These caveats highlight a major unmet need for more efficacious and faster‐acting treatments to improve the symptoms of MDD. In addition to antidepressants with different mechanisms, researchers are also interested in drugs that interact with nonselective, multitarget, or multiple ligands which can more effectively, rapidly, and broadly control the core symptoms of depression, as well treat comorbid symptoms such as pain, sexual dysfunction, cognitive impairment, weight gain, and insomnia.^12^ Agomelatine, which play the role of “synergism” as a MT_1_/MT_2_ receptor agonist and 5‐HT_2C_ receptor antagonist, alleviated depressive symptoms in both rodents and patients, while also showing anxiolytic effects, sleep‐promoting, and anti‐circadian desynchronization effects.^12^

Of particular importance, however, is the fact that currently more than 30% of depressed patients do not respond to first‐line treatment,^3^ whereas lower doses of ketamine confer a rapid antidepressant effect in these patients.^13^, ^14^ But its application is limited because of its psychotropic and addictive properties.^15^ In furthermore, recent studies also have suggested a number of pharmacological or non‐pharmacological ways to exert antidepressant‐like activity.^16^, ^17^

Consequently, agomelatine could represent a novel antidepressant with higher therapeutic efficacy. Studies have shown that in contrast to sexual dysfunction caused by SSRIs, treatment with agomelatine does not result in sexual dysfunction, possibly owing to overstimulation of 5‐HT_2C_ receptors.^18^ Moreover, this mechanism may explain the remission of anxiety symptoms in MDD. Evidence of other researches suggests that along with 5‐HT_2C_ receptors, gamma aminobutyric acid‐ergic neurons within the suprachiasmatic nucleus modulate the action of agomelatine and may also account for its anxiolytic effect.^19^, ^20^ Furthermore, studies have postulated that the synergistic effect of melatonin and 5‐HT_2C_ receptors underlies the antidepressant effect of agomelatine.^21^, ^22^

**FIGURE 1:**
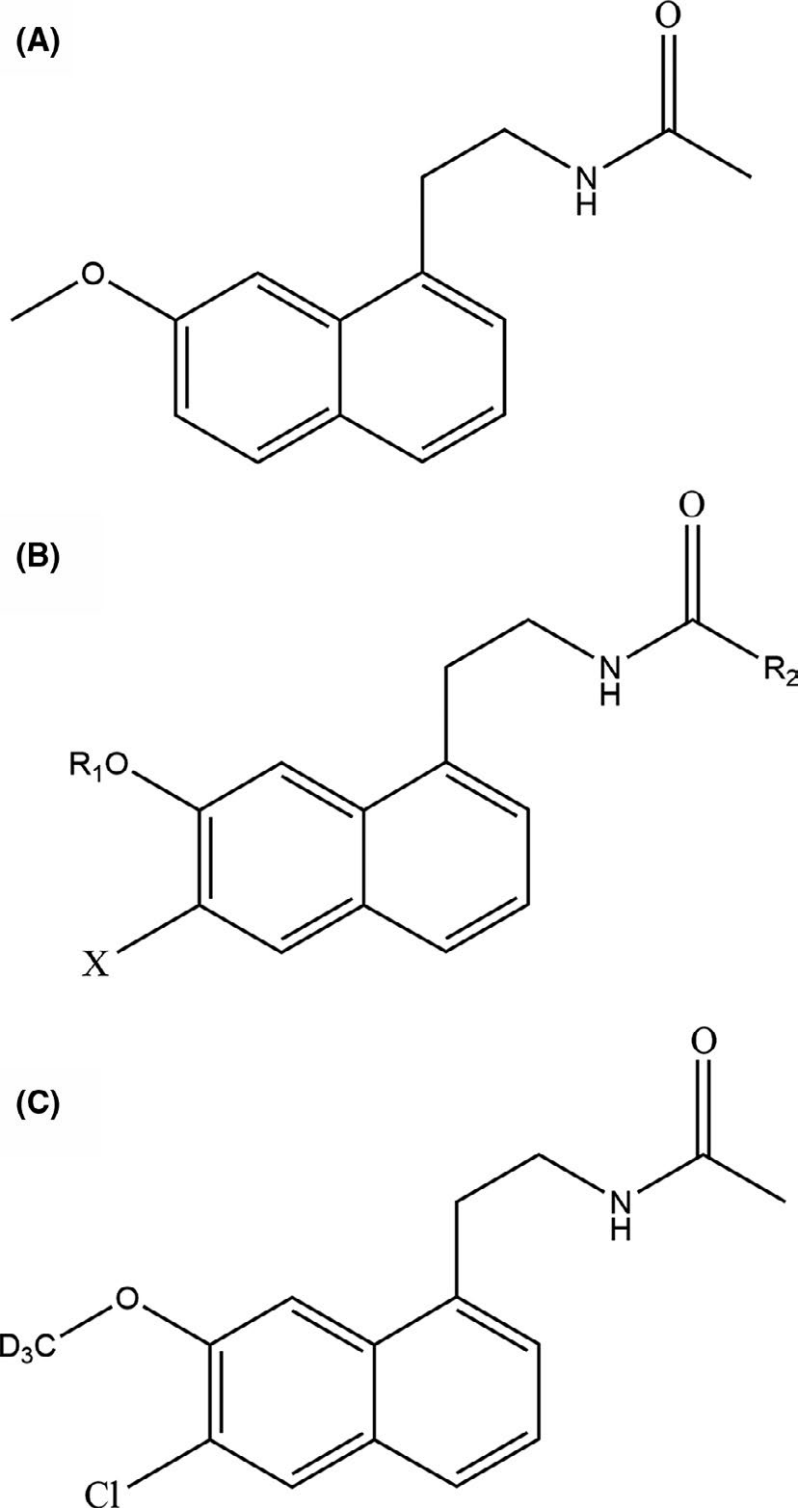
Chemical structure of agomelatine (A), structure of the series of derivatives of agomelatine (B), and GW117 (C)

Considering the factors given above, we designed and synthesized a string of compounds with new structures and screened the GW117 (Figure 1) a derivate compound of agomelatine with the same targets. Our previous study found that GW117 has lower toxic effects compared with agomelatine.^23^ In this research, we did the pharmacological characteristics of the GW117 detailed and believe that the results can provide a reference for progressing in phase II clinical trial in China.

## 2 MATERIAL AND METHODS

### 2.1 Animals

Male Sprague Dawley (SD) rats (165 ± 15 g) and ICR mice (20 ± 2 g) were supplied by the Experimental Animal Department of Capital Medical University (Beijing, China). Animals were housed in groups at a constant room temperature (23 ± 1°C), humidity (50–60%), and on 12‐h:12‐h light/dark cycle (8:00 am lighted). Food and water were available at all times. All procedures were in compliance with the guidelines for the care and uses of Laboratory Animals issued by the National Institutes of Health and were approved by the Animal Care and Use Committee of Capital Medical University (approval number SCXK‐2016–0002).

### 2.2 Chemicals

GW117 (purity >98.91%) and agomelatine (purity ≥99%) were both synthesized by Beijing GuangWei Pharmaceutical Technology Co., Ltd (Beijing, China). Fluoxetine, sodium carboxymethyl cellulose (CMC‐Na), 5‐HT, methyl lycaconitine, polyethyleneimine, bovine serum albumin, and phenylmethanesulfonylfluoride fluoride (PMSF) in the assay were purchased from Sigma. [^3^H]‐Lysergic acid diethylamide (LSD), [^3^H]‐melatonin, and OptiPhase SuperMix in the assay were purchased from Perkin‐Elmer Life Sciences (NEN). Human MT_1_/MT_2_ receptor membrane proteins were also Perkin‐Elmer products, namely ES‐620 (MT_1_) and ES‐621 (MT_2_). 5‐HT_2C_ receptor membrane protein was extracted from rat hippocampus and a stably transfected HEK293 cell line using a nucleus–cytoplasm–membrane preparation kit (Applygen Technologies Inc.,). The labeled ligand used in the assay was purchased from Perkin‐Elmer Life Sciences (NEN), while non‐labeled ligand was obtained from Sigma. Scintillation liquid was purchased from Perkin‐Elmer Life Sciences, and Folin‐phenol reagent was purchased from HAWI Science & Technology Co., Ltd. Each group was given suspension of the corresponding drug by gavage at 9 a.m. daily, the volume was 1 ml/100 g in rats or 0.1 ml/10 g in mice, and the control group was given the same volume of 0.5% CMC suspension.

### 2.3 Receptor binding assays

#### 2.3.1 Hippocampus membrane preparation

Hippocampal membrane preparations were obtained using previously described methods.^24^, ^25^, ^26^ Briefly, rats were decapitated and their brains were rapidly removed. Next, the hippocampus was dissected and subsequently homogenized in 40 volumes of ice‐cold buffer (50 mM Tris‐HCl Buffer, pH 7.4) and then centrifuged at 40,000 × *g* at 4°C for 10 min. The particles were re‐suspended and centrifuged again. In order to remove endogenous monoamines, the final suspension was incubated at 37°C for 20 min and then centrifuged as usual. The final pellets were frozen at −80°C at once for up to 1 week.

#### 2.3.2 Cell membrane preparation

Human MT_1_/MT_2_ receptor membrane proteins were obtained from Perkin‐Elmer (ES‐620 [MT_1_] and ES‐621 [MT_2_]). The stably transfected 5‐HT_2C_ receptor HEK293 cell line was prepared using a nucleus‐cytoplasm‐membrane preparation kit (Applagin Technologies Inc.). Briefly, cells were harvested at 4°C, 110 00 × *g* and centrifugation for 5 min. Homogenize pellets in the assay buffer (50 mM Tris‐HCl buffer containing 120 mM NaCl and 5 mM KCl, pH 7.4) and then centrifuged twice at 4°C, 36,000 × *g* for 15 min. Finally, pellets were re‐suspended in assay buffer and stored it at −80°C until it is used for in vitro experiments.

#### 2.3.3 MT_1_/MT_2_ receptor binding assay

GW117 or agomelatine was tested in competition binding experiments. Receptor membrane protein, MT_1_ or MT_2_ (10 μl), was added to all pipes. Non‐specific binding was determined by the presence of unlabeled 10 µM melatonin and pre‐reacted for 30 min. In test tubes, 30 μl of test drugs (MT_1_: concentration, 10^−4^–10^−10^ M; MT_2_: concentration, 10^−4^–10^−11^ M) was added. Additionally, 40 μl [^3^H]‐melatonin was added to all tubes. The volume in all reaction tubes was made up to 300 μl with Tris‐HCl buffer (50 mM, pH 7.4). Reactions were performed at 25°C for 1 h. The mixture was then spotted onto a type 49 glass fiber filter paper, suction filtered under vacuum, rinsed three times with 2 ml ice‐cold Tris‐HCl buffer (50 mM, pH 7.4), and dried by suction. Next, the filter paper was removed, dried by baking, and then put it in a scintillation bottle. Finally, 1 ml of scintillation fluid was added and then the radioactive intensity measured by liquid scintillation counting.

#### 2.3.4 5‐HT_2C_ receptor binding assay

GW117 or agomelatine was tested in competition binding experiments. Receptor membrane protein, 5‐HT_2C_ (50 μl), was added to all the tubes. Non‐specific binding was determined by the presence of unlabeled 10 μM 5‐HT and pre‐reacted for 30 min. In test tubes, 30 μl of test example compounds (concentration, 10^−4^–10^−10^ M) was added. Additionally, 40 μl [^3^H]‐LSD was added to all tubes. The volume in all reaction tubes was made up to 300 μl with Tris‐HCl buffer (50 mM, pH 7.4). Reactions were performed at 25°C for 1 h. The mixture was then spotted onto a type 49 glass fiber filter paper, suction filtered under vacuum, rinsed three times with 2 ml of ice‐cold Tris‐HCl buffer (50 mM, pH 7.4), and dried by suction. Next, the filter paper was removed, dried by baking, and then put it in a scintillation bottle. Finally, 1 ml of scintillation fluid was added and the radioactive intensity measured by liquid scintillation counting.

#### 2.3.5 [^35^S]‐GTPγS and [^3^H]PIP binding assays

This experiment was mentioned earlier.^27^ In short, HEK293 cell membrane stably expressing human 5‐HT_2C_ receptors or rat hippocampal tissue membrane or MT_1_/MT_2_ receptors was re‐suspended in buffer (50 mmol/L Tris, 100 mmol/L NaCl, 5 mmol/L MgCl_2_·6H_2_O, 0.1 mmol/L EDTA‐Na_2_·6H_2_O, 0.2 mmol/L EGTA, pH 7.4) and incubated with the test compound (100 μM GDP, 0.2 nM [^35^S]‐GTP, or [^3^H]PIP) for 60 min at 28℃. At the end of the experiment, Whatman GF/C filters pre‐soaked with distilled water were used for rapid filtration followed by washing with 5 ml of ice‐cold Tris buffer. Determination of non‐specific binding in the presence of GTPγS and radioactivity was measured by liquid scintillation counting. Calculation of the concentration produces a half‐maximal effect (EC_50_) and the maximal increase above the baseline value.

### 2.4 Animal behavioral tests

#### 2.4.1 Tail suspension test in mice

The tail suspension test (TST) was conducted as described earlier.^28^, ^29^ Eighty naive mice were randomly divided into eight treatment groups (*n* = 10/group). All mice received a single dose of the drug (p.o.). After 60 min of gavage administration, mice were suspended from the top of the apparatus (25 × 25 × 35 cm) using tape about 1 cm from the tail tip. The duration of immobility was recorded in the last 4 min for a total of 6 min. When mice are passively suspended immobile, they are judged to be stationary.

#### 2.4.2 Forced swimming test in rats

The forced swimming test (FST) was conducted as described earlier.^30^ Eighty naive rats were randomized into eight treatment groups (*n* = 9‐12/group). All rats received a single drug administration (p.o.). The procedure consisted of two sessions, to be specific, a pre‐test session and a test session. A cylindrical container (diameter, 20 cm; height, 40 cm; including 30 cm of water and kept at 25℃) was used. During the pre‐test phase, rats were required to perform forced swimming for 15 min. The test session was performed 24 h later, put the rats in the same cylindrical container for 5 min and immobility duration over 5 min recorded. Vehicle, agomelatine, or GW117 was given 1 h before the test. Rats were considered immobile when they entered a floating posture, specifically, immobile, passive, and with their heads above water.

#### 2.4.3 Locomotor activity in mice and rats

In order to confirm whether GW117 has an antidepressant activity, we used spontaneous activities in mice and rats to determine whether GW117 affects the central system. 64 ICR mice were randomly allocation to five therapy groups (*n* = 9‐12/group): control group, 5 mg/kg GW117, 10 mg/kg GW117, 20 mg/kg GW117, and 40 mg/kg GW117. All rats and mice received one administration (p.o.). After sixty minutes of gavage administration, each mouse or rat was located in the corner of an open field chamber (36 × 29 × 23 cm for mice; 76 × 76 × 46 cm for rats) to adapt to 5 min. The numbers of crossings and rearings were recorded during the subsequent 5 min.

#### 2.4.4 Chronic unpredictable stress model of rats

In order to further examine the antidepressant effects of GW117, chronic unpredictable stress (CUS) model was utilized; the methods were as described before.^29^, ^31^ After 1 week of training, rats underwent sucrose training of 48 h. After training, a sucrose baseline test was performed. Rats were randomly and evenly grouped depending on baseline of sucrose preference: vehicle (non‐stress), stress‐vehicle (distilled water), and stress‐fluoxetine (10 mg/kg), stress‐agomelatine (10, 20 or 40 mg/kg), and stress‐GW117 (5, 10, 20, or 40 mg/kg). Gastric gavage was given 1 h before the stress (08:00–09:00). Except for the non‐stressed group, all rats were stimulated by a series of stressors. stress methods included the following: fast on food and water (24 h), moisture cage (150 g sawdust bedding in 200 ml water), overnight illumination, low‐intensity strobe illumination (100 flashes/min), forced swimming (5 min at 10℃), white noise (110 dB), tail pinch (1 cm from tail root, 6 min), 45℃ cage tilt, and restraint (1–2 h). Stressors need to be used continuously and randomly. Non‐stressed rats received free food and water, but were required to fast for 14 h before sucrose preference test. After 4 weeks of stress, the sucrose preference test (SPT) (on day 25), open field test (OFT) (on day 28), and novelty‐suppressed feeding (NSF) test (on day 29) were carried out. A diagram of chronic unpredictable stress and behavioral testing was performed as shown in Figure 5.

#### 2.4.5 Open field test

The OFT device was a chamber (diameter, 122 cm; height, 45 cm) that was grouped equally into 16 sections. 24 h after the last administration, rats were put into the center of the chamber and the numbers of crossings and readings were recorded in 5 min.

#### 2.4.6 Sucrose preference test

The SPT was performed as previously reported.^32^ Rats were trained to administer sucrose water solution after 48 h fasting and water deprivation. Only 1% sucrose water was given for the first 24 h, and then 1% sucrose water and tap water were given for training at the end of 24 h. After training, feeding was performed for 3 days and a baseline measurement of sucrose drinking water was taken. Next, rats were fasted from food and water for 14 h. Rats were then made to select from two identical bottles for 1 h: one bottle with 1% sucrose solution and the other bottle with water. Sucrose and water intake were measured, and sucrose preference was calculated: SP =sucrose intake ×100%/ (sucrose intake +water intake). After 25 days of exposure to a stressful environment, the SPT was repeated to evaluate the drug effect.

#### 2.4.7 Novelty‐suppressed feeding test in the CUS model

The NSF test method performed as previously described.^33^ In summary, rats were fasted for 24 h and then put it into the corner of the chamber (76 × 76 × 46 cm) with the floor covered with 2 cm thick of sawdust and with 12 food pellets on it; the rats were allowed to explore freely for 5 min. Record the latency to eating pellets of rats. Eating food in rats refers to chewing or biting, but not by sniffing or playing with pellets.

#### 2.4.8 Learned helplessness paradigm

In order to demonstrate the antidepressant effect of sub‐chronic administration of GW117, ICR mice were acclimatized to the learned helplessness test, as reported previously.^34^ Ninety‐eight mice were randomly divided into no inescapable shock group (NIS, *n* = 10) and inescapable shock group (IS, *n* = 88). For 4 consecutive days, mice were putted into the shuttle box device (40 × 10 × 13 cm), the device was splited into two compartments, and mice could free access. In the IS group, each mouse received 360 inescapable shocks (current intensity 0.30 mA, duration 2 s, 3‐13 s variable interval, about 1 h) over four consecutive training days. Animals in the NIS group underwent the same handling without receiving the foot shocks. 10 trials were conducted on mice to screen helpless, each of which includes conditioned stimulation period (3‐s lamp on), non‐conditioned stimulation period (3‐s lamp +shock), and interval period (without any stimulus, 25 s). During the testing, the central gate is continuously opened, and the current intensity is 0.3 mA. If the mice did not pass through the central gate to avoid electric shock during the conditioned and non‐conditioned stimulus periods, it was considered escape failure (EF). Animals with EF less than or equal to 4 times were eliminated, and the remaining animals were divided into six groups according to the test results, which were the IS model, IS +fluoxetine (FLX, 10 mg/kg, ig.), IS +GW117 (0.625, 1.25, 2.5, 5 mg/kg, ig.) once a day for 4 days. The EF values were balanced for each group in the experiment. Using Graphic State software (Coulbourn Instruments Inc.,) recorded the number of escape failures and the latency to escape.

### 2.5 Statistical analysis

Differences between groups were determined by one‐way ANOVA and Dunnett's test. In all tests, differences with *p* < 0.05 were regard as significant difference. All the data are based on the mean ± S.E.M. and use GraphPad Prism 8 for analysis (GraphPad Software Inc.,).

## 3 RESULTS

### 3.1 5‐HT_2C_ receptor, MT_1_ receptor, and MT_2_ receptor radioligand‐binding assays

**FIGURE 2:**
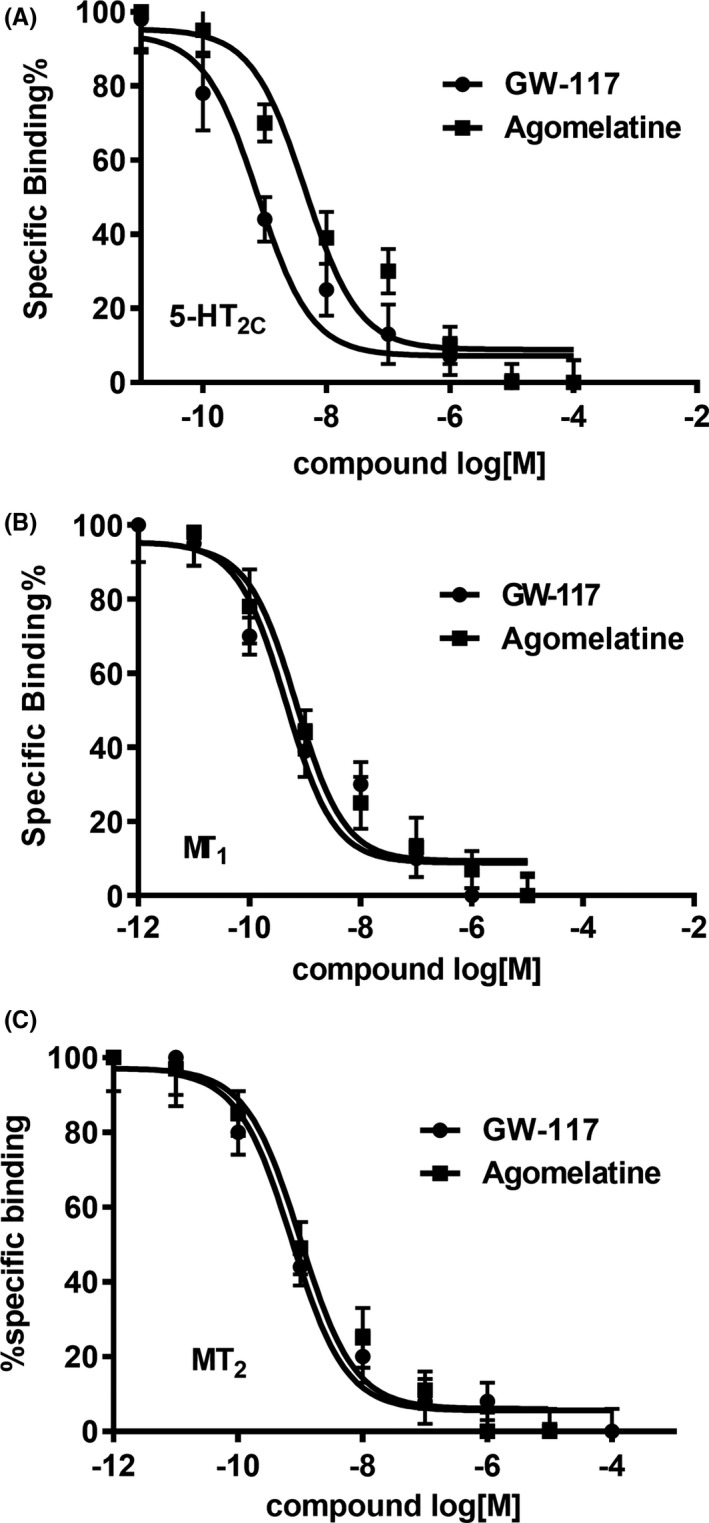
Receptor binding and function profile assays of GW117 and agomelatine. Ex vivo binding of [^3^H]‐LSD, [^3^H]‐melatonin to the 5‐HT_2C_ receptor in the hippocampus of rats (A) and MT_1_ receptor (B) and MT_2_ receptor (C)

Competition binding experiments with [^3^H]‐LSD and [^3^H]‐melatonin showed that GW117 has high affinity for 5‐HT_2C_ receptors, MT_1_ receptors, and MT_2_ receptors. The K_i_ value of GW117 was 0.32 ± 0.08, 0.12 ± 0.06, and 54 ± 7.31 nM, respectively (Table 1). Furthermore, the affinity of GW117 for the three targets was similar to that of agomelatine (Figure 2 and Table 1). Encouragingly, our agomelatine results are consistent with those reported in the literature.^35^

**TABLE 1:** The K_i_ values (nM) indicating the ability of GW117 and Agomelatine for the binding of [^3^H]‐LSD, [^3^H]‐melatonin. Data are presented as mean ± SEM. K_i_ values were calculated from three independent experiments on different days. Each concentration was run in triplicate

Drug	K_i_(Nm)		
	5‐HT_2C_	MT_1_	MT_2_
GW117	0.32 ± 0.08	0.12 ± 0.06	54 ± 7.31
Agomelatine	0.64 ± 0.25	0.09 ± 0.04	96 ± 14

### 3.2 [^35^S]‐GTPγS binding assay

**FIGURE 3:**
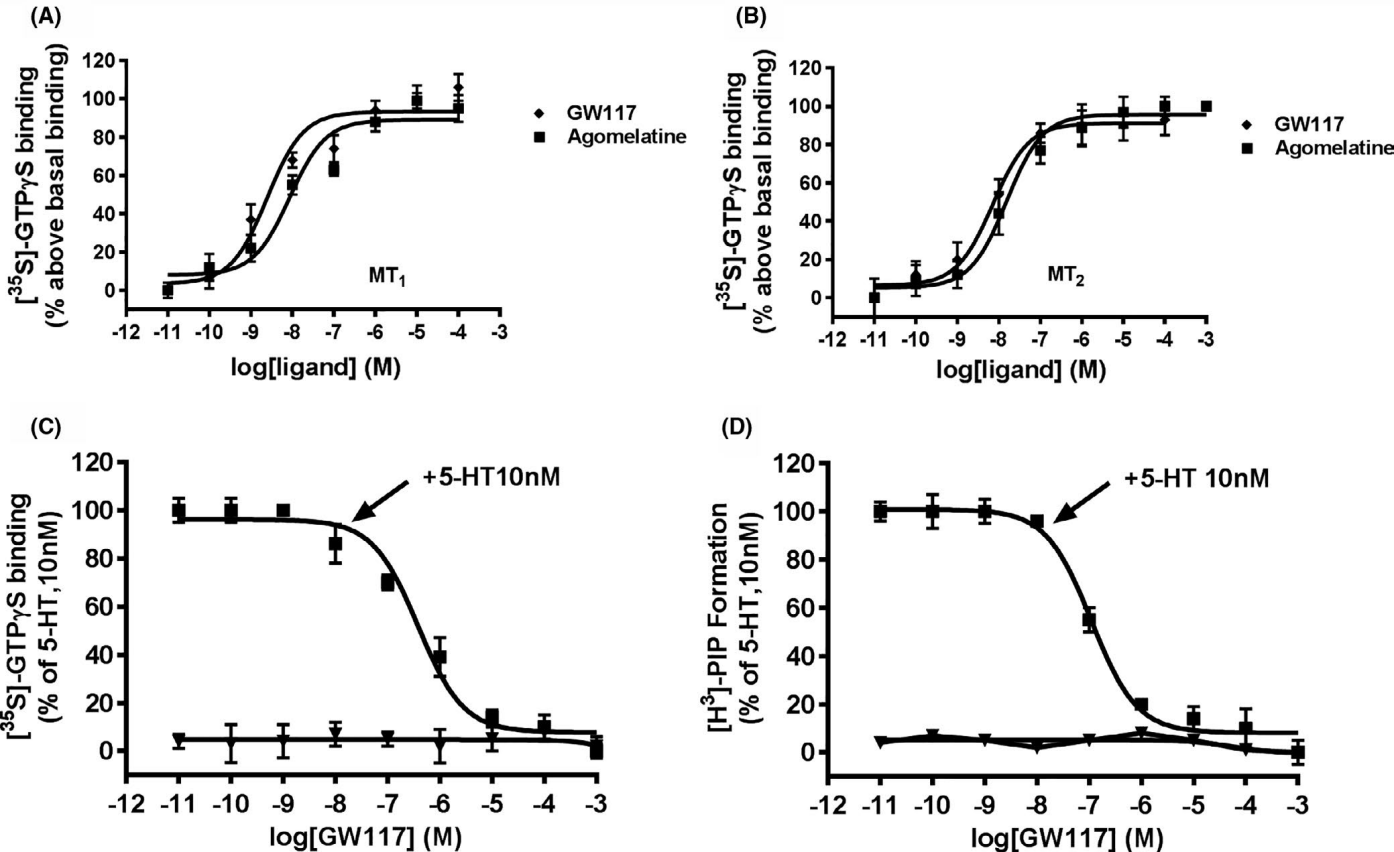
Effect of GW117 and agomelatine on [^35^S]‐GTPγS binding to MT_1_ receptor (A) and MT_2_ receptor (B), and 5‐HT_2C_ receptor from rat hippocampal membranes (C‐D). The results are expressed as the mean ± SEM. values of the percent of the respective basal binding obtained from 3 experiments performed in duplicate

The same target of agomelatine was used as a control to determine the conditions for the ligand‐stimulated [^35^S]‐GTPγS binding assay in this study. As shown in Figure 3A and B, GW117 stimulated specific [^35^S]‐GTPγS binding with an EC_50_ of 2.49 nM and 1.54 nM and a maximal increase over basal binding (% Emax) of 104 ± 7.34% and 103.2 ± 4.56% on the MT_1_ receptor and MT_2_ receptor, respectively. Agomelatine stimulated specific [^35^S]‐GTPγS binding with an EC_50_ of 9.49 nM and 15.47 nM and a maximal increase over basal binding (% Emax) of 94 ± 7.34% and 101.2 ± 4.56% on the MT_1_ receptor and MT_2_ receptor, respectively. The effect of GW117 on MT_1_ and MT_2_ receptors is consistent with the characteristics of a full agonist. Moreover, the activity and maximum agonistic potency of GW117 are similar to agomelatine. Furthermore, GW117 dose‐dependently blocked activation of [^35^S]‐GTPγS binding and [^3^H]PIP released by 10 nM 5‐HT (Figure 3C and D). These results suggest that GW117 is a typical 5‐HT_2C_ receptor antagonist. In summary, our results indicate that GW117 has high affinity for 5‐HT_2C_ receptors, MT_1_ receptors, and MT_2_ receptors, and is 5‐HT_2C_ receptor antagonist and MT_1_/MT_2_ receptor agonist.

### 3.3 Effect of GW117 in the TST in mice

**FIGURE 4:**
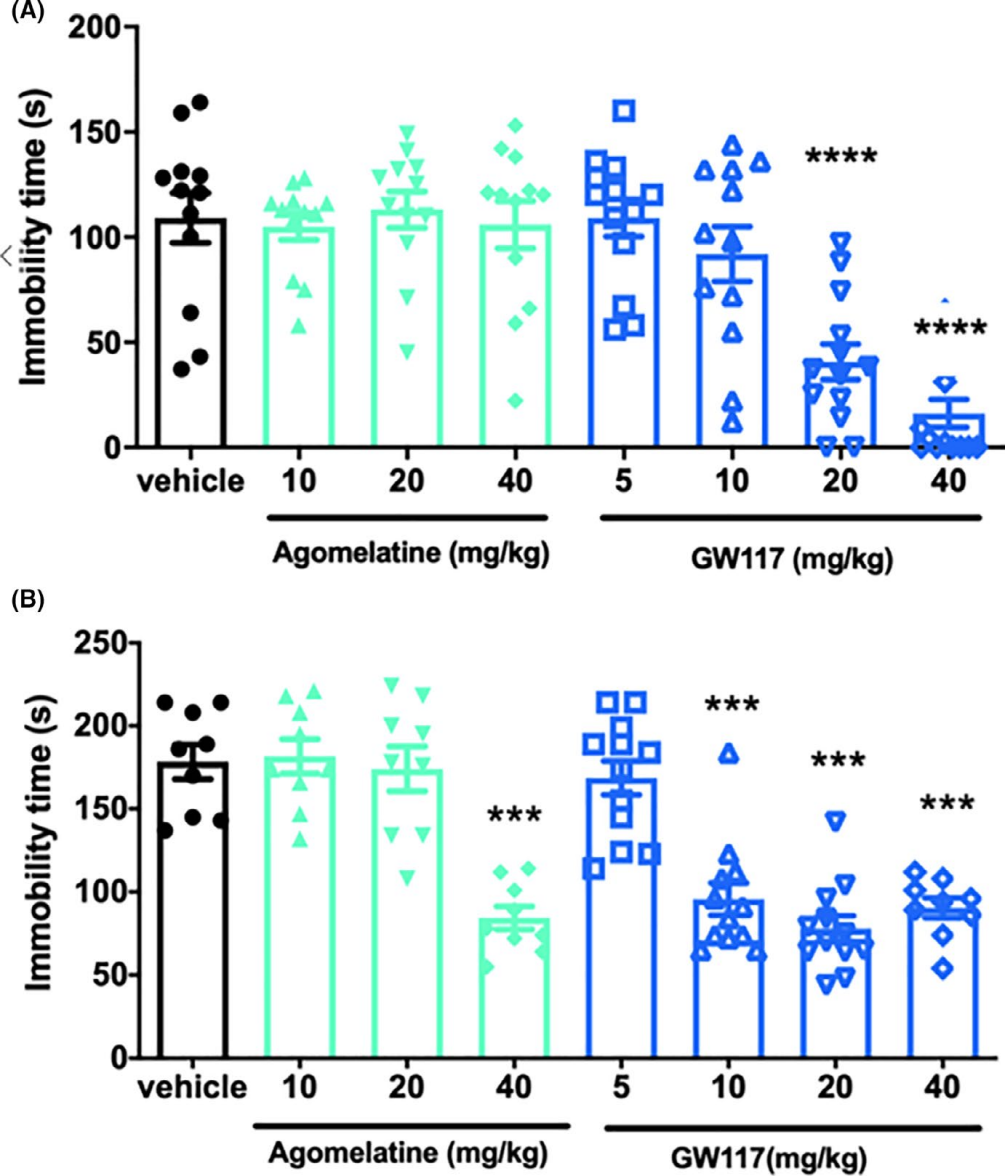
(A) The acute effects of GW117 (5, 10, 20, and 40 mg/kg, p.o.) on the immobility time in the TST in mice and the acute effects of GW117 on the immobility time in the FST in rats (B). Data are presented as mean ± SEM. (n = 8‐10/group). ***p < 0.001, ****p < 0.0001 vs. vehicle

Figure 4A shows the effect of GW117 (p.o.) on the immobility time of tail suspension test. As shown in Figure 4A, acute treatment with GW117 (20, and 40 mg/kg, p.o.) produced impact on the immobility time of tail suspension test in mice (one‐way ANOVA, *F*_[7, 91]_ = 15.36, *p* < 0.0001). Post hoc analysis showed that GW117 at the dose of 20 and 40 mg/kg markedly reduced the immobility time on TST test (Dunnett's test, ^***^ *p* < 0.0001 vs. vehicle). However, daily oral administration of agomelatine（10, 20 or 40 mg/kg） had no effect on immobility time. (*p* > 0.05 vs. vehicle; Figure 4A).

### 3.4 Effect of GW117 in the FST in rats

Figure 4B shows that GW117 (10 and 20 mg/kg, p.o.) significantly reduced immobility time in the force swimming test in a dose‐dependent manner (one‐way ANOVA, *F*_[7, 73]_ = 24.21, *p* < 0.0001). Post hoc analysis showed that GW117 at the doses of 10, 20, or 40 mg/kg markedly reduced the immobility time (Dunnett's test, ^***^p < 0.0001 vs. vehicle). Oral administration of positive drug agomelatine at 40 mg/kg significantly decreased the immobility time.

### 3.5 Locomotor activity in mice and rats

Table 2 describes the role of GW117 on spontaneous activity in mice and rats. In mice, GW117 (5, 10, 20, or 40 mg/kg) given by gavage had no effect on the number of crossings (one‐way ANOVA, *F*_[3, 44]_ = 0.04090, *p* = 0.9888) and rearings in the spontaneous activity test (one‐way ANOVA, *F*_[7, 92]_ = 0.5962, *p* = 0.7575). In rats, GW117 (5, 10, 20, or 40 mg/kg) given by gavage also had no effect on the number of crossings (one‐way ANOVA, *F*_[3, 35]_ = 0.1522, *p* = 0.9276) and rearings (one‐way ANOVA, *F*_[7, 76]_ = 0.3042, *p* = 0.9499).

**TABLE 2:** Effects of GW117 on locomotor activity in mice and rats. Data are presented as mean ± S.E.M. (*n* = 9‐12/group).

Species	Dose of GW117(mg/kg)	Locomotor activity	
		Crossing number	Rearing number
Mice	0	83.9 ± 5.5	20.5 ± 1.5
	5	86.8 ± 4.4	22.7 ± 1.8
	10	80.1 ± 4.8	22.6 ± 2.1
	20	83.1 ± 4. 4	20.7 ± 1.5
	40	86.3 ± 4.4	21.3 ± 1.4
Rats	0	62.1 ± 5.2	14.3 ± 0.8
	5	70.3 ± 5.1	14.2 ± 1.4
	10	71.3 ± 6.8	14.9 ± 1.5
	20	60.9 ± 4.4	13.8 ± 1.3
	40	63.5 ± 4.6	12.8 ± 1.3

### 3.6 Effect of GW117 on open field behavior in CUS rats

**FIGURE 5:**
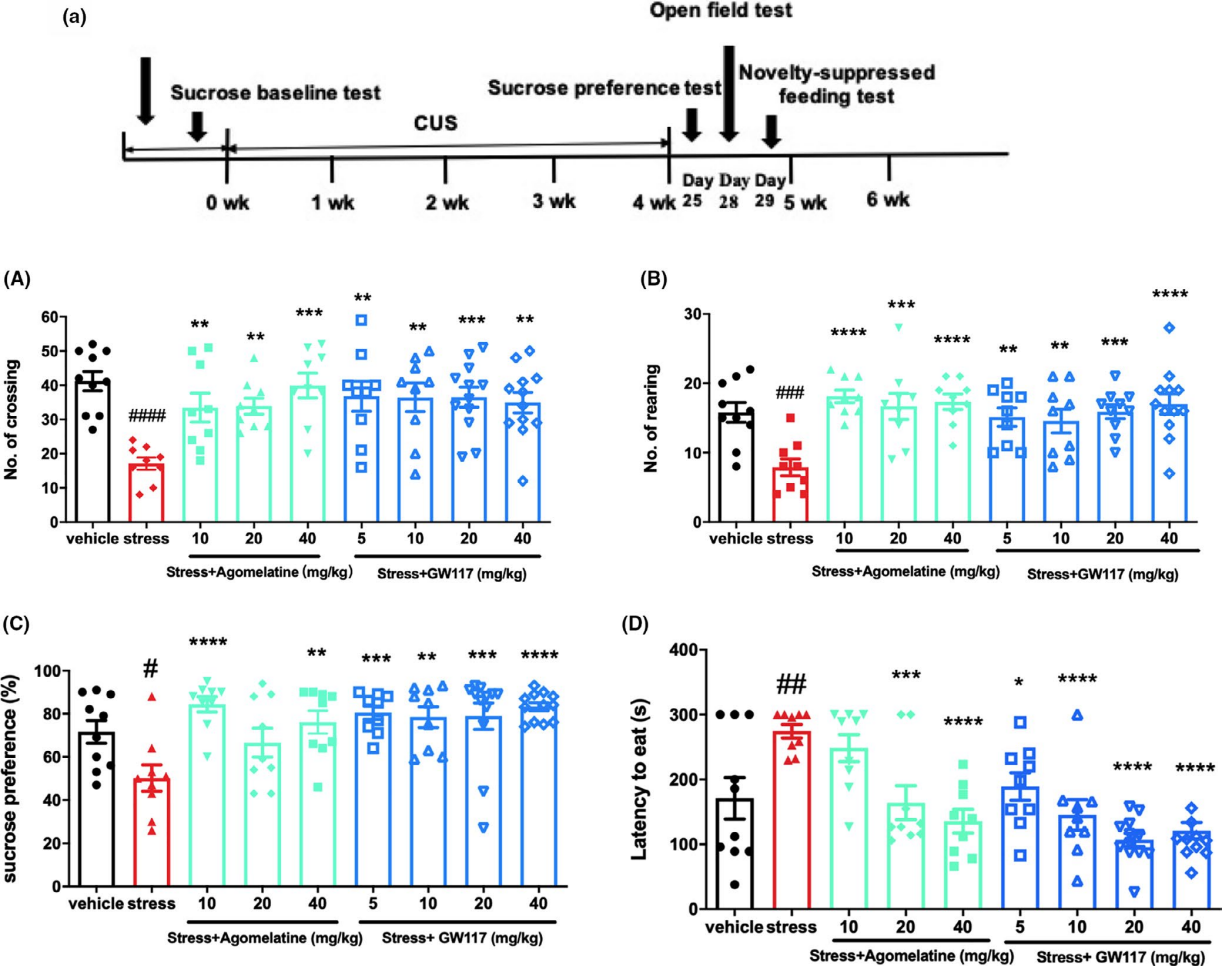
(a) The outline of design for chronic unpredictable stress and behavioral tests. Effects of GW‐117 (5, 10, 20, or 40 mg/kg), and agomelatine (10, 20, or 40 mg/kg) on the number of crossings (A) and number of rearings (B) in rats exposed to 4‐week stress procedure. (C) Effects of GW‐117 (5, 10, 20, or 40 mg/kg) and agomelatine (10, 20, or 40 mg/kg) on the sucrose preference in rats after 4‐week stress procedure. (D) Effects of GW‐117(5, 10, 20, or 40 mg/kg), and agomelatine (10, 20, or 40 mg/kg) on the latency to begin eating in rats exposed to 4‐week stress procedure. GW‐117 or agomelatine was administrated p.o. 60 min prior to stress procedure. Data are presented as means ± S.E.M. (n = 9‐12/group). **p < 0.01, ***p < 0.001, ****p < 0.0001 vs. stress, ^#^ *p* < 0.05, ^##^ *p* < 0.01, ^###^ *p* < 0.001, ^####^ *p* < 0.0001 vs. vehicle

As illustrated in Figure 5A and 5B, GW117 (5, 10, 20, 40 mg/kg) markedly add to the number of rearings and crossings. GW117 markedly add to the number of crossings (one‐way ANOVA with Dunnett's test, *F* (4, 46) =5.941, *p* = 0.0006) and rearings (one‐way ANOVA with Dunnett's test, *F* (4, 44) = 6.559, *p* = 0.0003) compared with CUS‐vehicle group.

### 3.7 Effect of GW117 on sucrose preference in CUS rats

As showed in Figure 5C, chronic administration of GW117 (5, 10, 20, and 40 mg/kg) markedly increased sucrose preference in stressed rats compared with the CUS‐vehicle group (one‐way ANOVA with Dunnett's test, *F* (4, 46) = 7.624, *p* < 0.0001).

### 3.8 Effect of GW117 on latency to begin eating in CUS rats

As illustrated in Figure 5D, chronic treatment with GW117(5, 10, 20 and 40 mg/kg) significantly reduced the latency to begin eating compared with vehicle‐stressed rats(one‐way ANOVA with Dunnett’s test, F (4, 46) = 18.04, *P* < 0.0001). A similar result was observed following agomelatine treatment (*F* [3, 32] = 11.35, *p* < 0.0001).

### 3.9 Antidepressant effect of GW117 on the learned helplessness paradigm in mice

**FIGURE 6:**
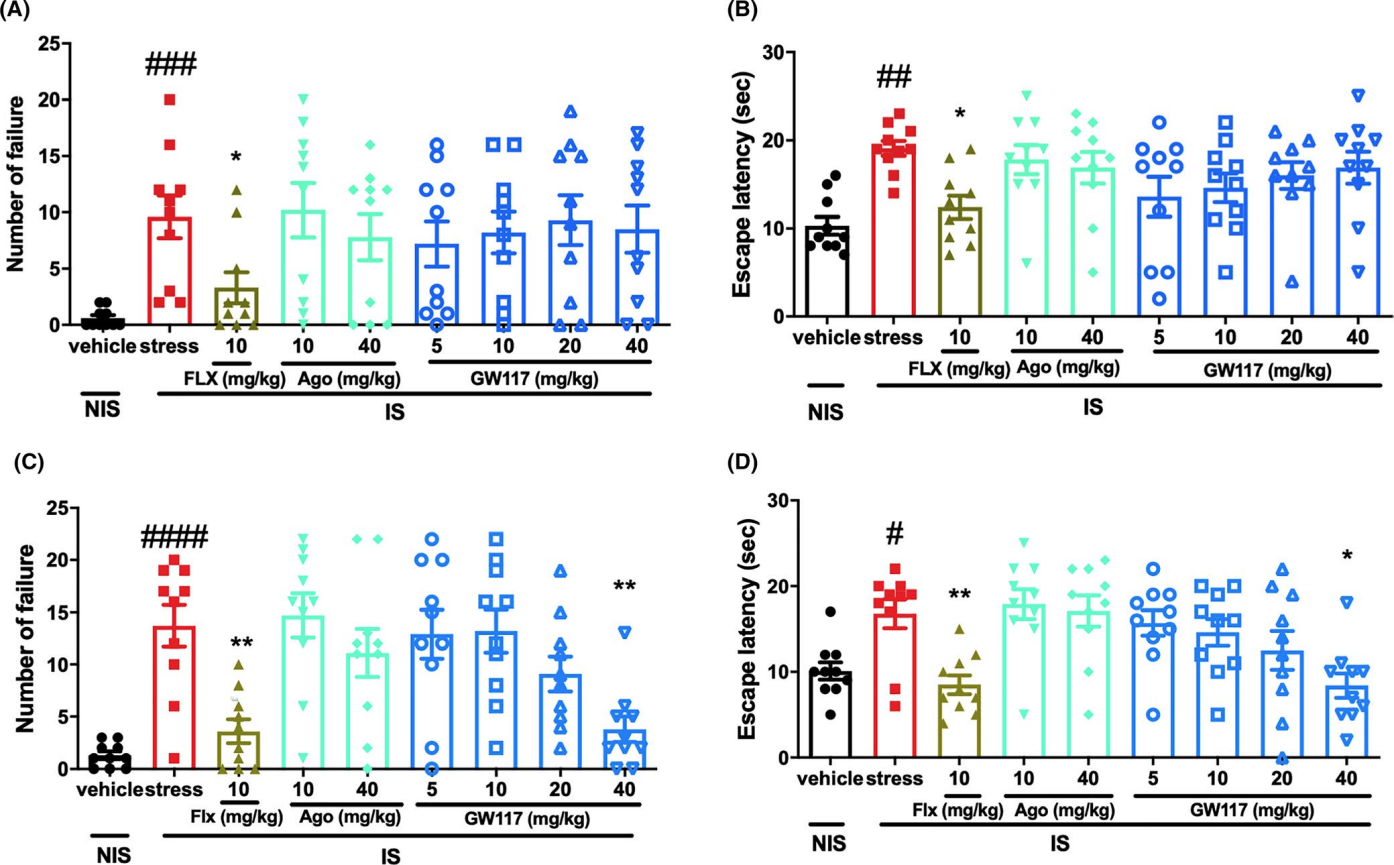
Effects of sub‐chronic administration of GW117 on learned helplessness paradigm in mice. The number of failures to escape and the escape latency on the second (A‐B) and fourth day (C‐D) after inescapable shock training. *n* = 10‐12/ group, ^####^ *<0.0001* *vs.vehicle group,* ^###^ *p* < 0.001 vs. vehicle group, ^##^ *p* < 0.01 vs. vehicle group, ^#^ *p* < 0.01 vs. vehicle group, **p < 0.01 vs. stress group, *p < 0.05 vs. stress group

The results are shown in Figure 6, after 4 consecutive days of inescapable shock training, it was found that mice in the IS group continued to show the deficit of avoidance behavior between the second day (Figure 6A and B) and fourth days (Figure 6C and D) after inescapable shock training. Compared to the NIS group, the number of escape failures and the escape latency significantly increased in the IS group. Sub‐chronic (4 days) administration of the positive drug Fluoxetine (10 mg/kg, ig.) after inescapable shock training significantly reversed the deficit of avoidance behavior and reduced the escape latency and the number of escape failures On the fourth day after inescapable shock training, sub‐chronic administration of GW117 significantly decreased the number of failures to escape at dose of 40 mg/kg (one‐way ANOVA with Dunnett's test, *p* < 0.001 vs stress for the 40 mg/kg dose) and decreased the escape latency at dose of 20 and 40 mg/kg (one‐way ANOVA with Dunnett's test, *p* < 0.05 vs stress for the 20 mg/kg dose and *p* < 0.01 vs stress for the 40 mg/kg dose), suggesting that GW117 has antidepressant effect on learned helplessness paradigm in mice. However, agomelatine has no effect on the number of failures and the escape.

## 4 DISCUSSION

In this research, we comprehensively evaluated the pharmacodynamics and pharmacology of GW117 and proved that GW117 is a new 5‐HT_2C_ receptor antagonist and melatonin MT_1_/MT_2_ receptor agonist with potential antidepressant‐like activity.

We investigated and compare the binding properties of GW117 to agomelatine at ES‐620 (MT_1_), ES‐621 (MT_2_), and 5‐HT_2C_ receptors in membrane protein from rat hippocampus and stably transfected HEK293 cells. GW117 displayed high affinity for MT_1_, MT_2_, and 5‐HT_2C_ receptors, was able to activate the activity of [^35^S]‐GTPγS on MT_1_ and MT_2_ membrane proteins, and dose‐dependently blocked the activation of [^35^S]‐GTPγS by 5‐HT. These results show that GW117 is a 5‐HT_2C_ receptor antagonist and an agonist of MT_1_ and MT_2_ receptors.

Here, we examined the antidepressant‐like potential activity of GW117 using behavioral despair paradigms. In the TST of mice, GW117 reduced immobility time by dose‐dependent manner and the minimal effective dose of 20 mg/kg. Then, the result of modified Porsolt FST in rats showed GW117 significantly reduced the immobility time at a dose of 10, 20, and 40 mg/kg, whereas agomelatine reduced the immobility time at a dose of 40 mg/kg. In the research, results in mice TST and rats FST experiments showed that GW117 exhibited better antidepressant effect compared with agomelatine, as the following by the lower minimal effective doses. Nowadays, immobility and swimming are seen as the expression of different coping strategies. Some studies suggest that drugs tend to improve depressive symptoms in humans and animals, such as anhedonia,.^36^, ^37^, ^38^

Since the stimulatory effect of antidepressants on the CNS (central nervous system) decreases the immobility time of the TST and FST, in order to eliminate false‐positive, we assessed the effect of GW117 on locomotor activity again. Encouragingly, GW117 had no effect on spontaneous activity in mice and rats. For further evaluating the antidepressant effect of GW117, the CUS model of rats has been successfully built to mimic the depressive‐like states which are the same as the clinical symptoms of depression.^29^ Here, four weeks of the stresses inhibited locomotor activity, decreased sucrose preference, and prolonged feeding latency in rats (all indicators of core symptom in major depression). Chronic administration of GW117 rehabilitation these symptoms to a normal state indicates that GW117 has an antidepressant effect, consistent with the acute effect. In addition, study has shown that the CUS model can also lead to anxiety behavior.^39^ The NSF test is commonly used to test the anxiolytic effects of drugs, and the model is thought to be sensitive to treatment with antidepressants.^33^ In our study, chronic administration of GW117 significantly shortened the feeding latency of the rats, compared with agomelatine (10 mg/kg) that was not effective in this test. To date, many laboratories have demonstrated that the learned helplessness model exhibits behavioral outcomes such as withdrawal and passive behaviors consistent with those exhibited by patients with major depressive disorder.^39^ We used this paradigm to investigate the antidepressant effect of GW117 and the results suggest that sub‐chronic treatment with GW117 has an antidepressant effect; the number of failures and escape latency was significantly reduced compared with the control mice. These findings were similar to those obtained using the first‐line SSRI antidepressant fluoxetine.

It should be noted that dose ranges for antidepressant‐like and anxiolytic effects of GW117 in rats and mice are between 5 and 40 mg/kg. Together with our present results, they provide support for the hypothesis that the antidepressant effects of GW117 may require combined action at both melatonin (MT_1_/MT_2_) and 5‐HT_2C_ receptors. In our subsequent study, we hope to elucidate the relationship between the modulation of depression by GW117 and the behavioral effects under stressful conditions.

Current antidepressants, including selective serotonin reuptake inhibitors (SSRIs), tricyclics, and monoamine oxidase inhibitors, have major limitations.^40^, ^41^ These agents must be continuously administered for a minimum of 2–4 weeks to produce therapeutic effects and only 30–40% of patients respond to first‐line treatment^42^, ^43^. Faster‐onset antidepressant treatments are greatly needed to improve the treatment of depression.^41^ Recent studies in rodents suggest that acute ketamine treatment induces rapid‐onset antidepressant effects through rapid activation of extracellular signal‐regulated kinase (ERK) and protein kinase B/Akt, which activate the mammalian target of rapamycin (mTOR) pathway.^41^, ^42^, ^43^ Early attempts of ketamine in the neurobiology of psychosis and schizophrenia were found to have a rapid antidepressant effect, and it was revealed that this rapid antidepressant‐like effect was associated with N‐methyl‐D‐aspartate (NMDA) receptors.^40^ John Krystal, Rob Berman, Dennis Charney, and colleagues at Yale University conducted a small, double‐blind, placebo‐controlled trial to test the antidepressant effects of ketamine.^42^ In this trial, a single dose of ketamine was observed to produce a rapid antidepressant response with a mean onset time of four hours lasting at least three days.^43^ Transient psychotomimetic and dissociative effects occurred after approximately one to two hours of treatment. But its use is limited due to the side effects of ketamine and the potential for drug abuse.^41^ Other work has shown that selective 5‐HT_2C_ antagonists are putative fast‐onset antidepressants. Five days of treatment with 5‐HT_2C_ antagonists induced antidepressant behavioral, molecular, and morphological effects that are comparable to those of current antidepressants and the fast‐acting agent ketamine.^40^, ^41^, ^42^, ^43^ Recently, other work has shown that acute ketamine treatment deactivates eukaryotic elongation factor 2 (eEF2) kinase, resulting in suppression of brain‐derived neurotrophic factor (BDNF) translation, which is required for onset of antidepressant behavioral effects.^41^ Therefore, GW117, a melatonergic agonist and selective 5‐HT_2C_ antagonist, maybe have a potential to induce faster therapeutic onset than SSRIs. In the following study, we will assess whether GW117 can induce faster‐onset antidepressant effects than current antidepressants using chronic models of antidepressant action.

## 5 CONCLUSIONS

In conclusion, our present results demonstrate that GW117 is a novel compound that acts as both a 5‐HT_2C_ receptor antagonist and a MT_1_/MT_2_ receptor agonist, and is likely a potent antidepressant in multiple animal models of depression. Our study offers new insights into the advance of antidepressants.

## CONFLICT OF INTEREST

Dr Jin has consulted for and received research funding from Guangwei Pharmaceutical Technology Co., Ltd., (Beijing, China). The remaining authors have nothing to disclose.

## AUTHOR CONTRIBUTIONS

ZJ designed the study, performed the behavioral tests, analyzed the data, and wrote the manuscript. NG and WG synthesized the novel compounds. TM contributed to the behavioral tests. XL and WZ contributed to the study design, data analysis, and manuscript revision.

## Data Availability

The data that support the findings of this study are available on request from the corresponding author. The data are not publicly available due to privacy or ethical restrictions.
